# Ragging: Let's say NO to it

**DOI:** 10.4103/0253-7613.51338

**Published:** 2009-04

**Authors:** Chetna Desai

**Affiliations:** Department of Pharmacology, BJ Medical College, Ahmedabad 380016, India. E-mail: chetna99@gmail.com

On 7^th^ March 2009, Aman Kachroo, 19, a first-year undergraduate student of Dr Rajendra Prasad Medical College complained to his parents about the brutal ragging on campus by completely drunk third-year students. He was beaten so badly that he died of brain hemorrhage. Sadly this was not the first such episode in an educational center in India. Amit Sahai, Durgesh Shukla, Manjot Singh, Chetan Raj, SP Manoj, C Abraham, Azad Nair, Kamlesh Sarkar, Mohan Karthik Tripathy are among the many victims of this sadistic and unhealthy practice carried out in name of “socializing” and “getting to know each other”! This is an obituary to that tender 19-year-old soul and several others who have become victims of ragging.

Ragging is a form of abuse of newcomers to educational institutions in several countries. It is similar to the American form, known as hazing, but is much more severe. Legally speaking ragging is an act which causes, or is likely to cause physical, psychological or physiological harm or apprehension or shame or embarrassment to a student. It includes teasing, abusing or playing practical jokes or causing hurt to any student or asking them to do any act, or perform anything which he/she would not, in the ordinary course, be willing to do. It started in its mild form in the 8th century AD during the Olympics in Greece. Later the armed forces of several countries and several student organizations in Europe and the USA started practicing this ritual. During World War I ragging underwent a massive transformation. Students who returned from the war and rejoined college brought with them the techniques of severe ragging practiced in army camps.

Ragging has reportedly caused at least 30-31 deaths in the last seven years in India, not all of which are those of freshers. C Lalitha, mother of Mukesh, ended her life due to the controversy surrounding the sexual abuse of her son during ragging. Three of the ragging deaths were those of seniors: two seniors were killed by a first-year student when he was being ragged; one senior ended his life when he was punished for ragging. With privatization of higher education in Indianone, academic institutions in India have been experiencing increasing raggingnone-related excesses.

There are certain myths associated with ragging. These myths have “glorified” and justified ragging. For example a popular myth is that ragging makes a student bold and prepares him for the difficult circumstances in life. The fact is that it is a weak acceptance of fate by the victim. It encourages exploitation and encourages non-resistant acceptance. It is also believed that ragging helps in breaking the ice between the seniors and freshers. It helps in their interaction and developing friendship between them. However, there can be more effective, enjoyable and healthy ways of interacting! The consequences of ragging are many. Freshers under severe stress may leave the system, or may suffer from serious psychological trauma and post-traumatic stress disorders. Occasionally, there may be physical injury, and some may even commit suicide. Promising careers are nipped in the bud.

Students are expected to cultivate the habit of understanding, sympathy and tolerance. Seniors are expected to guide the junior students to good campus life and must support them during their initial days. Instead there is a show of arrogance and supremacy over juniors. This arrogance, anger and hooliganism is not expected from the students. If such a character is carried over to professions, the society will suffer in the long run. The malaise, if not cured, may grow into a chronic incurable disease.

Educational institutes must take measures to sensitize students on the hazards of ragging. A policy of ‘Zero Tolerance” on ragging is essential. All students need to have a “Ragging Free” record as a prerequisite for completing their academic qualifications, for awards and for contesting college elections etc.

In India, resistance against ragging has grown only recently. Several states have passed legislation banning ragging, and the Hon'ble Supreme Court of Indianone has taken a firm stand to curb it. It has been declared a “criminal offence”. Spontaneous anti-ragging movements spearheaded by certain voluntary organizations have begun. These organizations conduct drives for public awareness and arrange for support to victims. Online groups like Coalition to Uproot Ragging from India (CURE), Stopragging and No Ragging Foundation are some of these. Among them, the No Ragging Foundation has transformed into a Non Government Organization and is registered as Society Against Violence in Education (SAVE), which is India's first registered antiragging nonprofit organization. These are the initiatives that could help curb ragging in the long run.

Parents must also take some responsibility and encourage their children to report any such incident immediately so that they can take up the issue with the management and prevent any tragic untoward outcome. Also they could counsel their wards to refrain from ragging.

Let there be “Aman” (peace). Aman lives and he is not dead for those people who bravely oppose ragging in campuses across India. Let there be an awakening in his name, bringing peace to those future students who will then fearlessly study in colleges.

